# In-Vitro Effect of Manuka Honey / Propolis Toothpastes on Bacteria and Biofilm Associated with Caries and Gingivitis

**DOI:** 10.3290/j.ohpd.c_1910

**Published:** 2025-03-27

**Authors:** Gert Jungbauer, Raphaela Lechner, Alexandra Stähli, Anton Sculean, Sigrun Eick

**Affiliations:** a Gert Jungbauer Department of Periodontology, School of Dental Medicine, University of Bern, Switzerland and Dental practice, Straubing, Germany. Wrote the original draft, contributed to the statistical analysis, discussed the results, read and approved the final manuscript.; b Raphaela Lechner Department of Periodontology, School of Dental Medicine, University of Bern, Switzerland. Performed the experiments in partial fulfillment of requirements for DMD, wrote the original draft, contributed to the statistical analysis, discussed the results, read and approved the final manuscript.; c Alexandra Stähli Private Docent, Department of Periodontology, School of Dental Medicine, University of Bern, Switzerland. Experimental design, discussed the results, proofread the manuscript, approved the final manuscript.; d Anton Sculean Professor and Chair, Department of Periodontology, School of Dental Medicine, University of Bern, Switzerland. Idea, discussed the results, proofread the manuscript, approved the final manuscript.; e Sigrun Eick Professor, Department of Periodontology (Laboratory of Oral Microbiology), School of Dental Medicine, University of Bern, Switzerland, idea, experimental design, contributed to statistical analysis, discussed the results, proofread the manuscript, approved the final manuscript.

**Keywords:** oral bacteria, oral hygiene, supragingival biofil

## Abstract

**Purpose:**

To investigate the antibacterial and anti-biofilm effects of two Manuka honey toothpaste formulations containing propolis (Manuka prop) or fluoride (Manuka F), in comparison with the toothpaste base (TP con) and a commercial toothpaste (TP com), on oral bacteria and biofilm.

**Materials and Methods:**

The minimum inhibitory concentration (MIC) of the formulations and controls were tested against five oral bacterial species. Both the effect on a multispecies dental biofilm precultured for 3.5 days as well as the inhibition of de-novo biofilm formation up to 24 h were investigated. Test substances at concentrations of 20%, 10% and 5% were applied to preformed biofilm for 1 min. The reduction in colony-forming units (cfu), metabolic activity, and biofilm mass were determined. Similarly, the test substances were applied to surfaces for 30 min before bacteria and media were added. The reduction of a tetrazolium dye (MTT assay) was used to assess cytotoxicity on gingival fibroblasts.

**Results:**

The MIC values of all toothpaste formulations including TP con were very low with the highest MIC of 0.04%. In precultured biofilms, both the number of colony forming units (cfu) and metabolic activity decreased following addition of any toothpaste. The greatest reductions of cfu were found after addition of 20% TP com (by about 6 log10) and after 20% Manuka prop (by about 2.3 log10). However, the biofilm mass was not reduced. Coating the surface with toothpaste formulation, the cfu in the newly formed biofilm decreased in a concentration-dependent manner, with TP com being most active. Both 20% of Manuka prop and Manuka F reduced the cfu counts more than the TP con at 24 h. The toothpaste formulations affected the viability of gingival fibroblasts in a concentration-dependent manner, with no differences observed among the formulations.

**Conclusion:**

The Manuka-honey containing toothpastes might be an alternative to toothpaste containing conventional chemical agents. Further research is needed to clinically examine the effect on caries and gingivitis prevention.

The oral cavity is inhabited by more than 1000 bacterial species. The survival strategy of bacteria in the oral cavity is to form multi-species biofilms. Bacteria in a biofilm increase their resistance to antibacterial agents about 1000x compared to planktonic, free-floating bacteria by the production of an extracellular matrix and cell-to-cell interactions.^
1,7,10
^ After 24 h, oral bacteria form a stable biofilm that evades chemical plaque control, and mechanical disintegration is needed to prevent further biofilm maturation and plaque-induced oral diseases, like caries and gingivitis.^
52
^ Therefore, home oral hygiene is recommended twice a day for at least two minutes using either a manual or electric toothbrush, as well as interdental cleaning devices.^
12
^ The use of a dentifrice did not increase plaque removal, but detergents may contribute to an antimicrobial effect of the product.^
42
^ Common detergents are, for example, sodium lauryl sulphate (SLS), sodium lauroyl sarcosinate, and cocamidopropyl betaine.^
19
^ The addition of fluoride compounds enhances the remineralisation of the enamel and is therefore recommended.^
12
^ Some detergents may have a cytotoxic effect on host tissue cells^
4,40
^ and should be viewed critically. Therefore, a growing interest in natural or organic toothpastes exists when comparing Google queries from 2004 to 2020.^
6,41
^ Potential natural detergents may be propolis and Manuka honey. Propolis or “bee glue” is a resinous mixture produced by honeybees consisting of saliva and beeswax. It is rich in biologically active compounds and shows antibiotic, antiviral, antimycotic and antioxidant properties. Active substances include: amino acids, enzymes, flavonoids, phenolic compounds, aromatic acids, esters, and terpenes. The antibacterial effect is due to increased membrane permeability caused by lipid interactions and disruption of the membrane potential. Propolis furthermore inhibits bacterial motility, protein synthesis and nucleic acid synthesis.^
30
^ Clinically, propolis was able to reduce bacteria associated with periodontal disease in periodontitis patients after a 3-month follow-up.^
17
^ In another study, propolis decreased *Streptococcus mutans *and further oral bacterial species after a 4 week period.^
31
^


Manuka honey is a monofloral honey produced by European honeybees (*Apis mellifera*) from the nectar of the Manuka tree, *Leptospermum scoparium*, and shows strong antibacterial activity.^
47
^ In contrast to non-Manuka honeys, the antibacterial property of Manuka honey is attributed to leptosperin, and predominantly to methylglyoxal (MGO).^
20
^ Manuka honey targets the bacterial cell wall, it inhibits cell division by downregulating the peptidoglycan hydrolase in Gram-positive bacteria, and destabilises the cell envelope by downregulating the outer membrane protein in Gram-negative species.^
15
^ Clinically, Manuka honey applied on teeth twice per day inhibited de-novo biofilm formation as effectively as 0.12% CHX within 72 hours.^
27
^ It significantly reduced salivary *S. mutans *counts when adjunctively used with mechanical cleaning over a 21-day period.^
27
^


The present study aimed to explore the activity of test toothpaste formulations containing Manuka honey and propolis or fluoride. In comparison with a standard commercial toothpaste, their effects on planktonic oral bacteria, the formation and destruction of a multi-species biofilm (resembling a supragingival biofilm), as well as their potential toxicity to gingival fibroblasts, were investigated.

## Material And Methods

### Test Products 

The following toothpaste formulations were included in the tests:

Toothpaste A (Mānuka Health New Zealand; Auckland, New Zealand), containing 4% Manuka honey and 0.2% propolis (Manuka prop)Toothpaste B (Mānuka Health New Zealand), containing 5% Manuka honey with 3200 ppm sodium fluoride (Manuka F)Toothpaste basis formulation (Mānuka Health New Zealand) containing xanthan gum, sorbitol, silica abrasive, stevia extract, tego betaine, colorant, demineralised water (formulation control; TP con)Toothpaste C: Colgate Total (Colgate-Palmolive; New York, NY, USA; positive control, TP com) containing glycerin, aqua, hydrated silica, calcium pyrophosphate, sodium lauryl sulfate, arginine, aroma, cellulose gum, zinc oxide, benzyl alcohol, poloxamer 407, zinc citrate, tetrasodium pyrophosphate, xanthan gum, cocaminopropyl betaine, sodium fluoride, sodium saccharin, phosphoric acid, sucraloseDistilled H_2_O (negative [growth] control; con).

The test products were provided by Mānuka Health New Zealand; TP com was bought in a Swiss supermarket.

### Test Strains 

The following bacterial strains were included in the experiments:

*Actinomyces naeslundii* ATCC 12104*Streptococcus gordonii *ATCC 10558*Streptococcus mutans *ATCC 25175*Streptococcus sobrinus *ATCC 33478*Fusobacterium nucleatum* ATCC 25586*Parvimonas micra* ATCC 33270*Campylobacter rectus *ATCC 33238*Prevotella intermedia* ATCC 25611*Tannerella forsythia *ATCC 43037*Porphyromonas gingivalis *ATCC 33277

Before experiments, the strains were precultivated on tryptic soy agar plates (Oxoid; Basingstoke, GB) containing 5% sheep blood (+ 10 mg/l N-acetyl-muramic acid [NAM; Merck; Darmstadt, Germany] in case of *T. forsythia*). Streptococci were cultured with 10% CO_2_, whereas agar plates with the other strains were incubated in an anaerobic chamber with the respective atmosphere (85% N_2_, 10% H_2_ and 5% CO_2_). Before use in the assays, the strains were suspended in 0.9% w/v NaCl according to McFarland 1.0.

### Determination of Minimal Inhibitory Concentrations

In the first part of the study, the minimal inhibitory concentrations (MICs) of the test substances and the controls were determined against five bacterial strains that are commensals (*S. gordonii *ATCC 10558) or associated with caries (*S. mutans *ATCC 25175) or periodontal diseases (*F. nucleatum *ATCC 25586, *T. forsythia* ATCC 43300, *P. gingivalis* ATCC 33277).

Of the toothpaste substances, a two-fold dilution series was prepared starting from 40% in dH_2_O. 100 µl was pipetted into each well of a 96-well plate. Then, 90 µl of double concentrated test medium (adjusted Mueller-Hinton broth for streptococci and Wilkins-Chalgren broth supplemented with 10 µg/ml β-NAD [Merck] +NAM for *T. forsythia*), and finally 10 µl of bacterial suspension were added. For *S. gordonii *and S. mutans, bacterial growth was recorded after 18 h of incubation in a CO_2_ atmosphere, and bacterial growth of the other strains was recorded after 24 h of incubation in an anaerobic atmosphere by measuring absorption at 600 nm in a plate reader. In addition, subcultivation of the suspension was performed.

The MIC was defined as the lowest concentration without visible turbidity (or with clear growth inhibition).

The experiments were performed in independent replicates.

### Effect on Precultivated Multi-species Biofilm

The experimental design represented the application of the toothpaste without intensive brushing. Biofilms were cultured from bacteria 1-9 (without *P. gingivalis*) for 3.5 days in three 96-well plates each. First, the wells were coated with 10 µl of protein solution (1.5% BSA, 0.67% mucin)/well for 10 min. Meanwhile, the strains were adjusted to McFarland 0.5 in 0.9% w/v NaCl. Two parts of strain 1 were mixed with one part of strain 2 and each 4 parts of strains 5-9. This bacterial suspension was added in a ratio of 1:9 to nutrient broth (Wilkins-Chalgren broth with 10 mg/ml NAM + 20 mg/l β-NAD). 225 µl were pipetted into each well.

After two days of anaerobic incubation, 25 µl of a bacterial suspension prepared as before but containing only strains 5 to 9 were added again. After 3.5 days, the media were removed, the biofilms were washed briefly, and then 20 µl of the test substances in concentrations of 20%, 10%, 5% (diluted in dH_2_O with 1.5% BSA) were added to the biofilms in each well. After 1 min of exposure, 180 µl of nutrient broth were added and the biofilms were incubated for 1 h before being analysed.

Analysis included three aspects. The remaining biofilm was scraped from the surface and mixed by pipetting. The total number of colony forming units was determined by sub-cultivation on agar plates and incubation. Further, the metabolic activity was quantified by using resazurin and the total biofilm quantity (bacteria and matrix) was measured by crystal violet staining.^
32
^


### Effect on Multi-species Biofilm Formation

The experimental design represented the application of the toothpaste after intensive brushing. Four 96-well plates were coated with 20 µl of the test substances in three concentrations (20%, 10%, 5%) for 30 min. Then 10 µl of a proteinaceous solution (1.5% BSA, 0.67% mucin) were added for 10 min, before nutrient broth (Wilkins-Chalgren broth with 10 mg/ml NAM + 20 mg/l β-NAD) containing bacterial mixture (bacteria 1-9) and prepared as described above was added. One well-plate was incubated for 4 h, and the other three for 24 h. At 24 h, analysis was performed as described above; at 4 h, only colony-forming units were counted.

### Cytotoxicity of Toothpastes on Gingival Fibroblasts

Gingival fibroblasts were harvested from patients undergoing an esthetic periodontal surgery. The patients had been informed about the use of their cells in research and gave written consent. According to the guidelines, no previous approval from the Cantonal ethics committee KEK was necessary, as the biomaterials were categorised as “irreversibly anonymised”. The gingival fibroblasts (2nd – 4th passage) were handled as described before.^
7
^ After aspirating the cell culture media from the confluent monolayer and after a two-fold washing with PBS, the test substances were added in concentrations of 1.25%, 2.5%, 5% and 10%. After 10 min of exposure, the MTT (3-(4,5-dimethylthiazol-2-yl)-2,5-diphenyltetrazolium bromide) tetrazolium colorimetric assay^
25
^ was applied as a measure of cell viability.

### Statistical Analysis

All biofilm experiments were conducted in at least two series with six independent replicates (12 values) per test substance. Cytotoxicity tests were performed with cells obtained from two different donors in two independent experiments with four replicates each. Statistical analysis was performed with the help of software (SPSS 29.0, IBM; Armonk, NY, USA) using ANOVA with a post-hoc Bonferroni test.

## Results

### Minimal Inhibitory Concentrations of Toothpastes Against Selected Oral Species

All obtained MIC were very low, with the highest obtained MIC being 0.04% (Table 1). Even the toothpaste formulation without active ingredients was very active.

**Table 1 table1:** Minimal inhibitory concentration (MIC) of the different toothpaste formulations against selected oral bacteria

	Manuka prop	Manuka F	TP con	TP com
Streptococcus gordonii ATCC 10558	≤0.02%	≤0.02%	≤0.02%	≤0.02%
Streptococcus mutans ATCC 25175	0.04%	≤0.02%	0.04%	≤0.02%
Fusobacterium nucleatum ATCC 25586	≤0.02%	0.08%	≤0.02%	≤0.02%
Tannerella forsythia ATCC 43037	≤0.02%	≤0.02%	≤0.02%	≤0.02%
Porphyromonas gingivalis ATCC 33277	≤0.02%	≤0.02%	≤0.02%	≤0.02%


### Precultured Multi-species Biofilms

**Fig 1 fig1:**
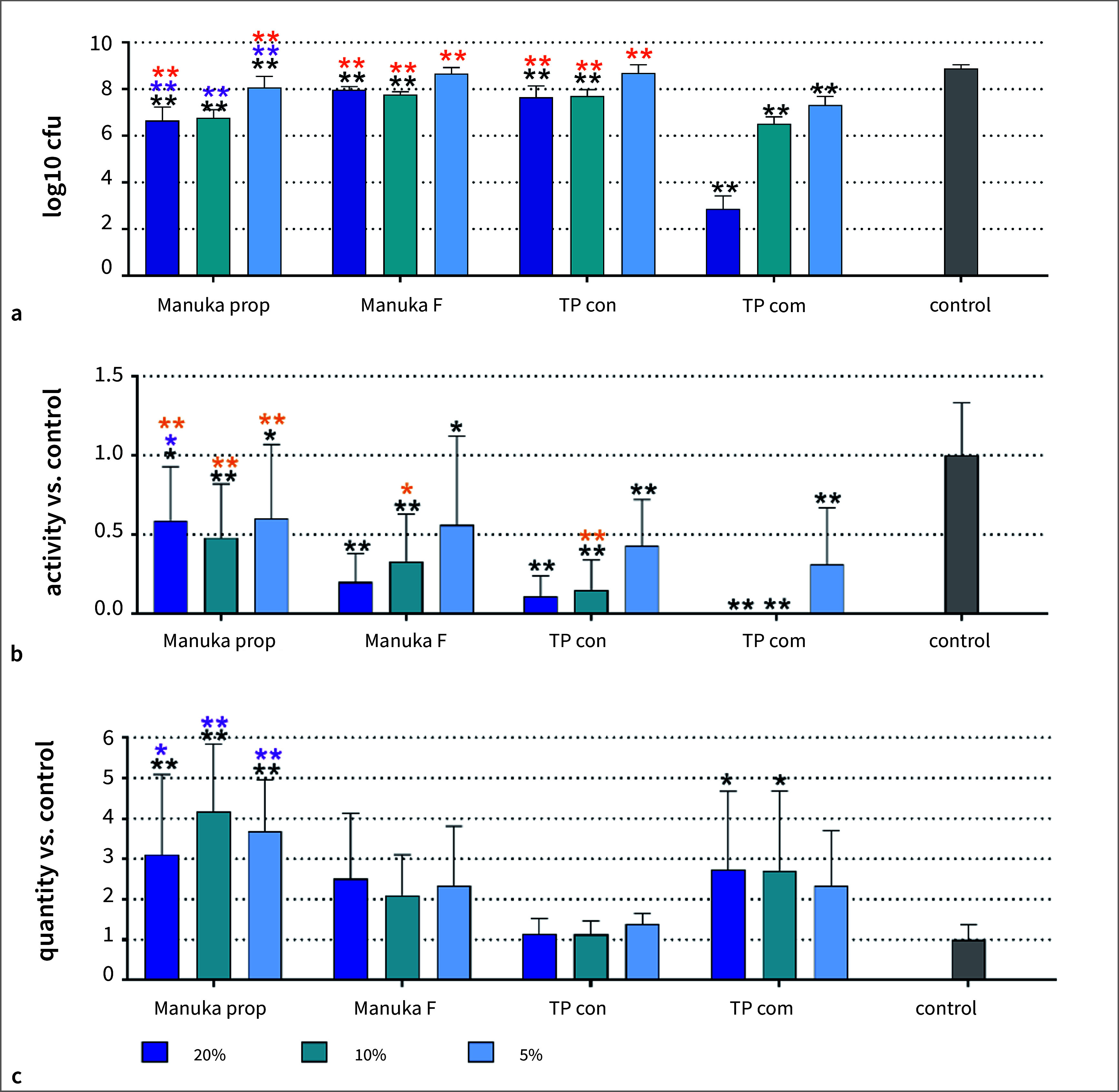
Total counts (a), metabolic activity (b), and biofilm “mass” (c) of a 3.5-day-old 9-species biofilm without (control) and with different concentrations of two Manuka toothpaste preparations (Manuka prop, Manuka F), a toothpaste basis without active ingredients (TP con) and a commercial toothpaste (TP com). p < 0.05, **p < 0.01 vs control. *p < 0.05, **p < 0.01 vs the respective concentration of TP con. *p < 0.05, **p < 0.01 vs the respective concentration of TP com.

The total number of cfu in the 9-species biofilm without any added toothpaste formulation was 8.89±0.16 log10. After the addition of any toothpaste, the cfu counts decreased (Fig 1a). The highest decreases were found after addition of 20% TP com (by about 6 log10 to 2.87 log10) and after 20% Manuka prop (by about 2.3 log10 to 6.66 log10). Except for 5% of the TP con and Manuka F, all other results were statistically significant compared to the control (all p < 0.001). When comparing within the different concentrations of the toothpaste formulations, it was apparent that the addition of propolis to the Manuka toothpaste statistically significantly decreased the number of cfu vs TP con (5% p = 0.003; 10%, 20% p < 0.001); the addition of fluoride did not seem to have an effect. The number of cfu with Manuka F was very close to that of TP con.

Regarding metabolic activity, the general results were similar: each formulation statistically significantly decreased the metabolic activities of the biofilms compared to the control (all concentrations of TP con and TP com, 10%, 20% Manuka F, 10% Manuka prop, p < 0.001; 5%, 20% Manuka prop, 5% Manuka F, p < 0.05; Fig 1b). Again, the effect of TP con was similar to that of the Manuka F. However, unlike the cfu counts, the effect was less pronounced for Manuka prop vs TP con (20% p = 0.019). Regarding biofilm mass, there was no visible reduction; in contrast, higher values were found after applying Manuka prop (each p < 0.001) and higher concentrations of TP com (10% p = 0.025, 20% p = 0.022) vs the control.

### Inhibition of Biofilm Formation

**Fig 2 Fig2:**
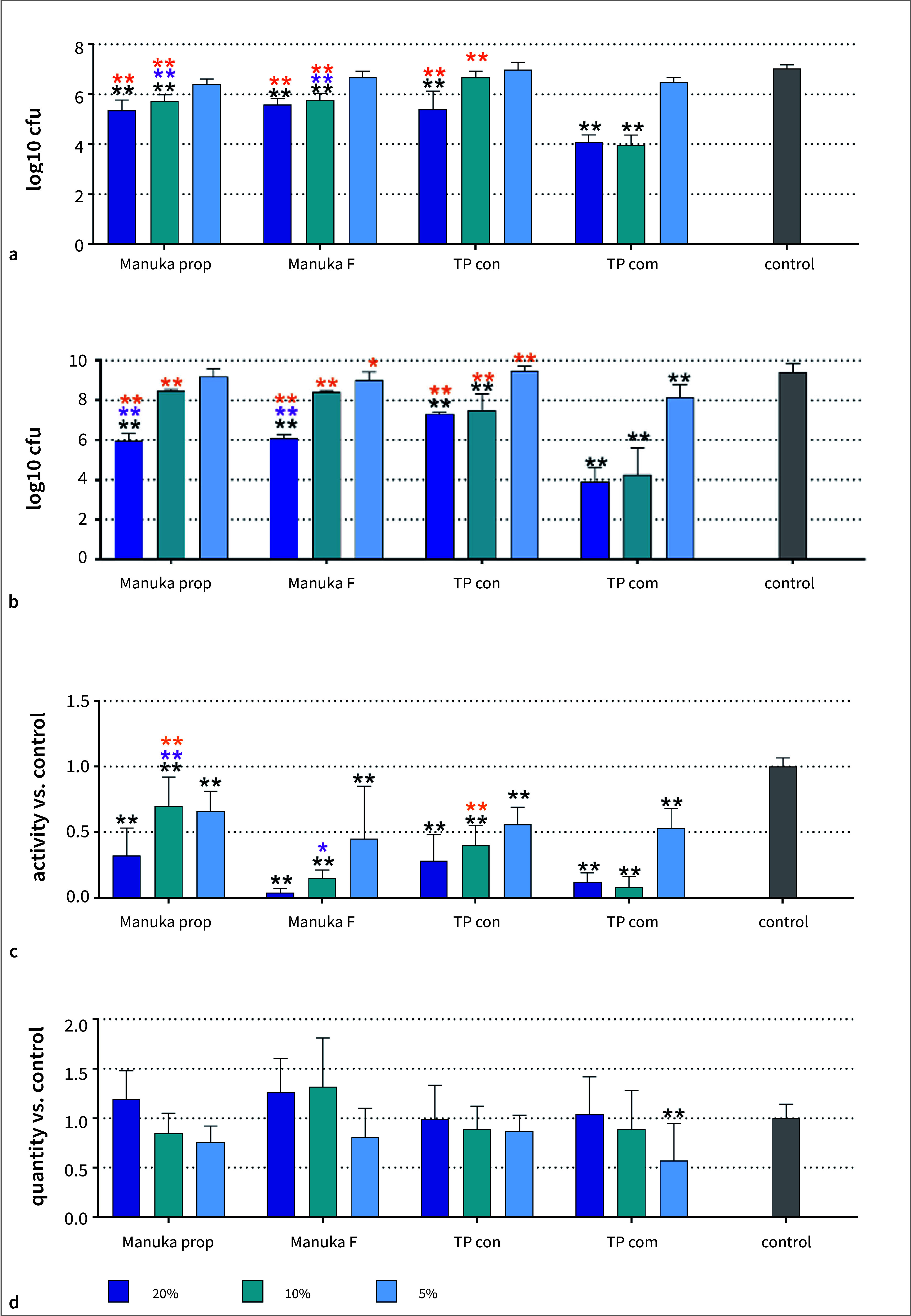
Total counts after 4 h (a) and 24 h (b), metabolic activity (c), and biofilm “mass” (d) after 24 h of a newly formed 9-species biofilm without (control) and after coating the surface with different concentrations of two Manuka toothpaste preparations (Manuka prop, Manuka F), a toothpaste basis without active ingredients (TP con) and a commercial toothpaste (TP com). **p < 0.01 vs control. *p < 0.05, **p < 0.01 vs the respective concentration of TP con. *p < 0.05, **p < 0.01 vs the respective concentration of TP com.

The total number of cfu in the 9-species control biofilms reached 7.03±0.15 log10/well after 4 h and 9.41±0.43 log10 after 24 h of incubation. When the the surface was coated with a toothpaste formulation, the cfu decreased in a concentration-dependent manner (Figs 2a and 2b). The differences vs the control were always statistically significant when a 20% formulation was applied (p < 0.001 at 4h and 24 h), and most often statistically significant for a 10% formulation (TP com, Manuka prop at 4 h and 24 h, Manuka F at 4 h, TP con at 24 h, p < 0.001). At 4 h and 24 h, the highest decreases were found after adding 10% and 20%TP com (by about 3 log10 at 4 h and 5.52 – 5.26 log10 after 24 h), while 20% Manuka prop and Manuka F reduced the log10 cfu by about 1.65 log10 at 4 h and by about 3.5 log10 after 24 h. Within the 10% and 20% concentration of the toothpaste formulations, the biofilms after TP com had lower cfu counts compared to the other formulations (p < 0.001 at 4h and 24 h). Twenty percent of Manuka prop and Manuka F reduced the number of cfu more than TP con at 24 h (both p < 0.001).

In terms of the metabolic activity at 24 h, each formulation statistically significantly decreased the metabolic activities of the biofilms vs the control (p < 0.001 vs control; Fig 2b). The 20% TP com and 20% Manuka F were the most effective; the effect of TP con was similar to that of Manuka F. However, in contrast to the cfu counts, the effect was less pronounced for Manuka prop vs TP con (10% p = 0.019). Regarding biofilm mass, there was only a minor change according to formulation, showing that only 5% TP com reduced the biofilm mass vs the control (p < 0.001; Fig 2d).

### Viability of Gingival Fibroblasts (Fig 3)

The toothpaste formulations affected the viability of the gingival fibroblasts in a concentration-dependent manner. The viability of the cells was statistically significantly lower after applying any 5% and 10% formulation vs the control (10% TP com p = 0.006, all others p < 0.001). Within the concentrations, there was no statistically significant difference among the formulations.

## Discussion

The present study investigated the effect of test toothpaste formulations containing Manuka honey and propolis or fluoride on oral bacteria as a “supragingival” biofilm as well as on the viability of gingival fibroblasts. The effect of the test toothpaste formulations was compared with the basis formulation and a commercially available toothpaste. The test formulations demonstrated strong antibacterial activity, partially disrupted existing biofilms, and inhibited biofilm formation. At higher concentrations, cytotoxicity could not be excluded and was comparable to that of the commercial toothpaste. The results highlight different aspects of the antibacterial and anti-biofilm properties of the tested formulations: the MIC against single planktonic bacteria, the activity against a preexisting biofilm and the potential to inhibit the de-novo biofilm formation. The evaluation of the anti-biofilm activities included the reduction of cfu, decreased metabolic activity as well as total biofilm mass. To increase the reproducibility of the experiments, a multi-species biofilm model with a defined composition of bacteria was used. A limitation of this study may be that the mechanical effects of toothbrushing were not considered in the experimental setup. The obtained MIC values demonstrated high antibacterial activity against planktonic oral species. A species-specific dependence was not observed, and commensals (S. gordonii) were also affected. The values are comparable to results for commercial toothpastes.^
3,23
^


Biofilm-reducing activities were found for all toothpaste formulations, including TP con. With respect to the list of ingredients provided by the manufacturer, antibacterial active agents in TP con could be stevia^
24
^ and sorbitol.^
28,37
^


The supplementation of the TP con with Manuka honey/fluoride or Manuka honey/propolis further increased the anti-biofilm activity of TP con. Both test formulations inhibited the biofilm formation to a greater extent than TP con. Thus it can be suggested that this effect is related to the Manuka honey. Manuka honey is known for its antibacterial activity against cariogenic and periodontal bacteria,^
9,33,34
^ for instance, the formation of a 6-species oral biofilm was completely inhibited by 200-500 µg/ml Manuka honey.^
9
^


The Manuka F formulation decreased the metabolic activity but not the number of cfu of the biofilms in comparison with the Manuka prop formulation. To exert a direct effect on bacterial counts in biofilm, high concentrations of 2000 ppm are needed, as shown in an single-species *S. mutans *biofilm.^
14
^ According to the manufacturer, the Manuka F formulation contains 3200 ppm F^-^ and exceeds the concentration of most of the commercially available dentifrices. In the experiments, the formulation was diluted to a final concentration of 640, 320 and 160 ppm which obviously did not affect bacterial counts in the multi-species biofilm. The decrease of metabolic activity in the biofilms might be related to an effect on bacterial enzymes. It is known that fluoride inhibits activities of a variety of bacterial enzymes, e.g., enolase, heme catalases, ureases, phosphatases, and glucosyltransferases; the latter is important in biofilm matrix synthesis.^
14,46,48
^


In the preformed biofilm, the reduction of cfu was more pronounced by Manuka prop compared to Manuka F and TP con. This is of interest as the concentration of Manuka honey in that formulation is lower than those with fluoride, and the concentration of propolis with 0.2% (according to the manufacturer’s information) is relatively low. Propolis is well known for its antibacterial activity against oral bacteria.^
13,51
^ Red Brazilian propolis in similar concentrations reduced bacterial counts by about 1 log10 and in addition reduced the metabolic activity of a multispecies biofilm.^
5,22
^ In a study performed at our laboratory, European and Brazilian propolis significantly reduced the cfu in an established biofilm by up to 6 log10 cfu, and effectively inhibited the de-novo biofilm formation.^
38
^ There, the concentration of the propolis extracts was 10%.^
38
^ Another study using a 10% propolis toothpaste in vitro found an antibacterial effect against* S. mutans, Staphylococcus aureus,* and *P. gingivalis* comparable to that of 0.2% CHX.^
39
^ On the other hand, using a toothpaste containing 0.9% propolis resulted in no reduction of cfu counts in a multi-species biofilm model.^
44
^ All these studies underline that the anti-biofilm activity of propolis is strongly dependent on the concentration used.

In case of the TP com, the anti-biofilm/antibacterial activity can also be related to the chemical detergent sodium lauryl sulphate.^
35
^ Other ingredients may also contribute, e.g., benzyl alcohol has been described as destabilising bacterial membrane structures.^
50
^ Oral healthcare products containing cocamidopropyl betaine had an antibacterial activity,^
26
^ although the anti-biofilm effect might be negligible.^
16
^ Our result of the strong antibiofilm effect of TP com confirms the findings of another study, where TP com exhibited increased antibiofilm properties compared to products with natural ingredients.^
18
^


All tested formulations exhibited concentration-dependent cytotoxicity.^
4
^ This is in accordance with the literature, where most toothpastes exert a cytotoxic effect.^
4,11
^ For TP com, it might be related to its detergent sodium lauryl sulphate^
4
^ and cocamidopropyl betaine.^
40
^ In concentrations up to 40% and exposure time of 30 min, Manuka honey did not exert a clear cytotoxic effect against keratinocytes and fibroblasts.^
49
^ In contrast, even low concentrations of propolis (about 0.05%) decreased cell viability of epithelial cells and gingival fibroblasts after 48 h of exposure.^
21
^ The results for cytotoxicity were obtained in an experimental set-up using cell monolayers and are not directly transferable to the in-vivo situation. The cytotoxicity in a mono-layer model is higher than in more complex three-dimensional models^
36
^ and complex tissues. Furthermore, the exposure time of toothpaste can be expected to be much shorter. Despite some in-vitro toxicity of toothpastes, there is no evidence of the supplemented propolis and/or honey being cytotoxic. Regarding the natural products propolis and Manuka honey, their safety profiles are positive for long-term use.^
2,45
^ However it should be noted that contact allergies to propolis might occur.^
29
^


Nevertheless, it remains to be noted that aside from toothpaste, the mechanical disintegration of the biofilm is a conditio sine qua non. To remove the dental biofilm, an appropriate toothbrush together with an adequate brushing technique should be applied.^
43
^


## Conclusion

Toothpaste formulations containing Manuka honey/propolis or Manuka honey/fluoride may represent a natural alternative to commercially available products containing chemical detergents with regard to antibacterial properties and cytotoxicity. Natural ingredients like Manuka and propolis may contribute to biofilm management at home and therefore to the prevention of caries and gingivitis. However, the obtained in-vitro results need to be confirmed in clinical studies. The concentrations of Manuka honey and propolis used in this study may be somewhat low. A higher antibacterial effect could likely be achieved by increasing the concentrations of these natural products.

## Acknowlegdements

The authors are grateful to Eric Nabil Risse (Department of Periodontology, Laboratory of Oral Microbiology, University of Bern) for his excellent assistance in performing the in-vitro assays. This study was funded by Mānuka Health New Zealand Ltd., Auckland, New Zealand. The funder had no role in the final design of the study, the collection, analyses, or interpretation of data, writing the manuscript, or the decision to publish the results.

## References

**Fig 3 Fig3:**
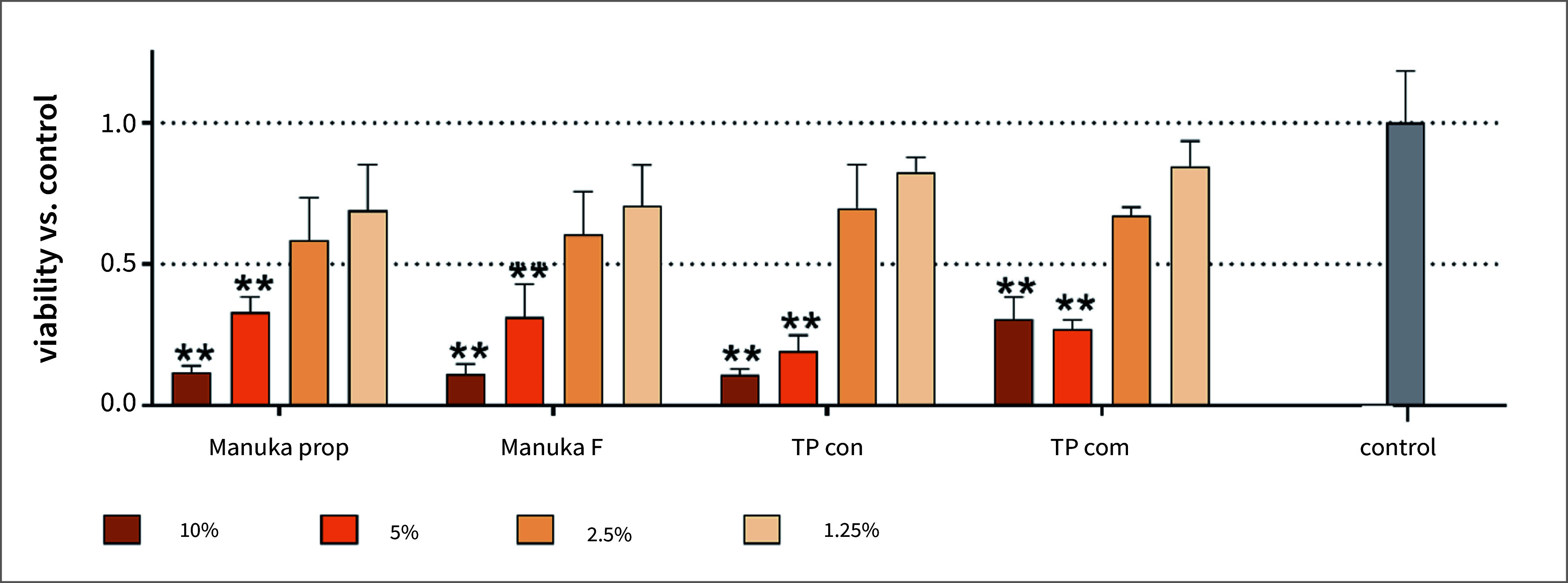
Viability of gingival fibroblasts after 10 min exposure of different concentrations of two Manuka toothpaste preparations (Manuka prop, Manuka F), a toothpaste basis without active ingredients (TP con) and a commercial toothpaste (TP com). **p < 0.01 vs control.
